# Human pluripotent stem-cell-derived alveolar organoids for modeling pulmonary fibrosis and drug testing

**DOI:** 10.1038/s41420-021-00439-7

**Published:** 2021-03-15

**Authors:** Jung-Hyun Kim, Geun Ho An, Ji-Young Kim, Roya Rasaei, Woo Jin Kim, Xiong Jin, Dong-Hun Woo, Choongseong Han, Se-Ran Yang, Jong-Hoon Kim, Seok-Ho Hong

**Affiliations:** 1 Department of Internal Medicine, School of Medcine, Kangwon National University, Chuncheon, 24341 South Korea; 2 Department of New Drug Development, NEXEL, Co., Ltd, Seoul, South Korea; 3 Laboratory of Stem Cells and Tissue Regeneration, Department of Biotechnology, College of Life Sciences and Biotechnology, Science Campus, Korea University, 145 Anam-ro, Seongbuk-gu, Seoul, 02841 Korea; 4 Department of Thoracic and Cardiology, School of Medicine, Kangwon National University, Chuncheon, 24341 South Korea

**Keywords:** Pluripotent stem cells, Respiratory tract diseases

## Abstract

Detailed understanding of the pathogenesis and development of effective therapies for pulmonary fibrosis (PF) have been hampered by lack of in vitro human models that recapitulate disease pathophysiology. In this study, we generated alveolar organoids (AOs) derived from human pluripotent stem cells (hPSCs) for use as an PF model and for drug efficacy evaluation. Stepwise direct differentiation of hPSCs into alveolar epithelial cells by mimicking developmental cues in a temporally controlled manner was used to generate multicellular AOs. Derived AOs contained the expected spectrum of differentiated cells, including alveolar progenitors, type 1 and 2 alveolar epithelial cells and mesenchymal cells. Treatment with transforming growth factor (TGF-β1) induced fibrotic changes in AOs, offering a PF model for therapeutic evaluation of a structurally truncated form (NP-011) of milk fat globule-EGF factor 8 (MFG-E8) protein. The significant fibrogenic responses and collagen accumulation that were induced by treatment with TGF-β1 in these AOs were effectively ameliorated by treatment with NP-011 via suppression of extracellular signal-regulated kinase (ERK) signaling. Furthermore, administration of NP-011 reversed bleomycin-induced lung fibrosis in mice also via ERK signaling suppression and collagen reduction. This anti-fibrotic effect mirrored that following Pirfenidone and Nintedanib administration. Furthermore, NP-011 interacted with macrophages, which accelerated the collagen uptake for eliminating accumulated collagen in fibrotic lung tissues. This study provides a robust in vitro human organoid system for modeling PF and assessing anti-fibrotic mechanisms of potential drugs and suggests that modified MGF-E8 protein has therapeutic potential for treating PF.

## Introduction

Pulmonary fibrosis (PF) is a fatal chronic respiratory disease characterized by accumulation of myofibroblasts and deposition of extracellular matrix, leading to respiratory failure^1^. To date, only two drugs, Pirfenidone (PFD, Esbriet) and Nintedanib (Nib, Ofev), have been approved for human PF based on the demonstration of a reduction in lung function decline^2^. No survival advantage was noted and the injured lungs did not recover following use of these drugs, highlighting the need to identify novel and effective therapeutic drugs for the treatment of PF^3,4^.

One of major obstacles for the development of effective therapies for PF has been a lack of in vitro human models that recapitulate features of alveolar tissue and the pathophysiology of the disease. While human primary pulmonary alveolar epithelial cells (pAECs), comprised of alveolar type 1 and type 2 epithelial cells (AEC1 and AEC2), are the most representative of the in vivo situation, low accessibility, a limited proliferation capacity, and loss of functional properties over time remain a bottleneck for their use in PF studies^5^. An immortalized pulmonary epithelial cell line (A549) derived from a human pulmonary adenocarcinoma has been widely used instead of pAECs to model the alveolar epithelium for biopharmaceutical research and to evaluate the pulmotoxicity of suspected harmful materials; however, these cells respond to toxins differently and exhibit altered phenotypic, genetic, and functional properties compared with primary pAECs^6–8^. Alternatively, primary pAECs from other laboratory animals can be used, but species-specific differences in molecular mechanisms of fibrosis and responses to potential drugs may also limit applicability to humans^9,10^. Importantly, recent studies suggest that three-dimensional configurations, such as alveolar organoids (AOs) and spheroids, offer several advantages compared to conventional two-dimensional pAEC monolayer cultures for studying early lung development, modeling disease, and screening for novel drugs^11–15^. Therefore, the development of an in vitro human AO model using a reliable and renewable biological source to generate pAECs that phenotypically and functionally resemble primary pAECs offers great promise for PF modeling and drug screening.

The ability to differentiate human pluripotent stem cells (hPSCs) into three germ layer lineages offers promise for the development of in vitro three-dimensional organoids that recapitulate the complexity and functions of in vivo tissues^16^. In the lungs, hPSCs have been successful employed to generate several organoids that represent different respiratory compartments, such as proximal and distal airways and alveoli^11–15^. More recently, hPSC-derived alveolar and airway organoids with genetic defects associated cystic fibrosis, surfactant deficiency, and impaired surfactant secretion have been generated to support studies to understand the role of specific cell types during pathological development^11,17–19^. These previously reported organoids, however, have not been employed for evaluation of the therapeutic potential of novel drugs for the treatment of PF.

In the present study, we generated hPSC-derived multicellular AOs composed of functional AEC1, AEC2, alveolar progenitor cells (AEPs) and mesenchymal cells. These AOs exhibit phenotypic and genetic resemblance to in vivo human alveolar tissues and recapitulate critical PF pathological features, including inflammation, fibrosis, and collagen accumulation, following transforming growth factor (TGF)-β1 treatment. We further evaluated these fibrotic AOs for their use in evaluation of the therapeutic efficacy of novel drugs for the treatment of PF.

## Results

### Generation of multicellular AOs from hPSCs

We have previously reported efficient, reproducible, and stepwise alveolar epithelium differentiation from hPSCs, which not only generate functional AECs but also mesenchymal support cells^6^. By taking advantage of this protocol, two-dimensional cultures of emerging distal alveolar cell types (*SOX9*, *ID2*, *SFTPB*, and *SFTPC*)^20^ on day 21 of differentiation were dissociated to single cells to form uniform aggregates by forced aggregation (Supplementary Fig. 1A). The aggregates were transferred into AEC maturation medium and cultured for 6 days to establish AOs, which exhibited an alveolar sac-like structure with multiple alveoli and layers of epithelial cells (Supplementary Fig. 1B). Immunofluorescence staining showed that hPSC-derived AOs express markers of AEPs (EPCAM, CPM, and NKX2.1), AEC1 (AQP5 and T1α), AEC2 (SFTPC), and mesenchymal cells (VIMENTIN) (Supplementary Fig. 1C). Moreover, AEPs, AEC1, AEC2, and mesenchymal cell-related genes were robustly expressed in AOs compared to undifferentiated hPSC cultures according to quantitative PCR (qPCR) analysis (Supplementary Fig. 1D). These observations confirmed that our method enables the generation of multicellular AOs containing AEPs, AEC1, AEC2, and mesenchymal support cells from hPSCs.

### Modified MFG-E8 protein (NP-011) ameliorates TGF-β1-induced fibrotic changes in hPSC-derived AOs via suppression of ERK signaling

**Fig. 1:**
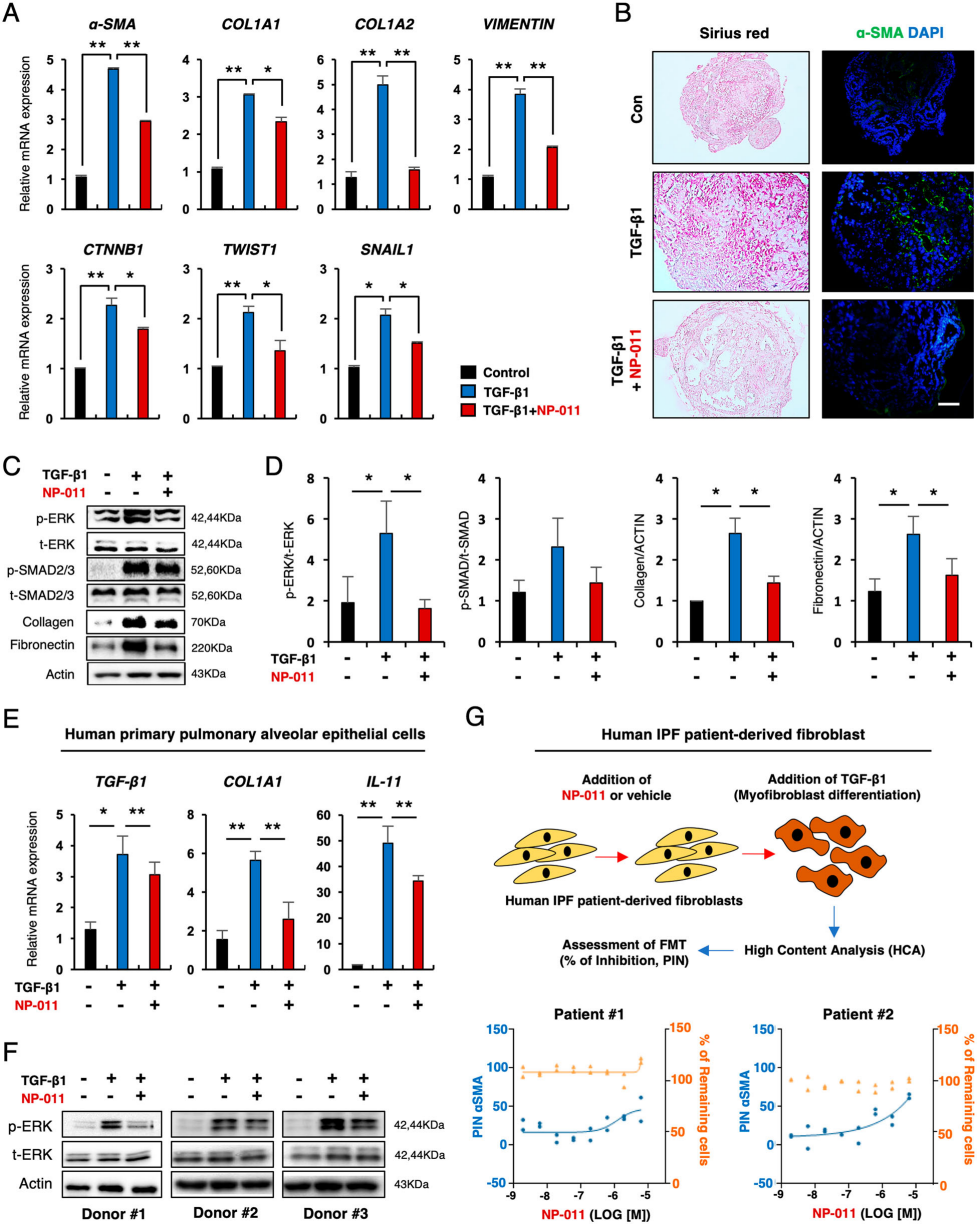
NP-011 reduces TGF-β1-induced fibrosis in AOs. **A** qPCR of the indicated fibrosis-related genes in control AOs and AOs with TGF-β1 (25 ng/ml)-induced fibrosis. **B** Representative images show Sirius red and α-SMA staining of AO sections from the indicated groups. Scale bars, 100 μm. **C**, **D** Western blotting (**C**) and subsequent quantification of p-ERK, p-SMAD2/3, and Collagen (**D**) in AOs from the indicated groups. Actin was used as a loading control. **E** qPCR analysis for expression of *TGF-β1*, *COL1A1* and *IL-11* from the indicated groups. **F** Western blot analysis for p-ERK in human pAECs incubated with TGF-β1 (25 ng/ml) or TGF-β1 and NP-011 (500 ng/ml) for 72 h. Actin was used as loading control. **G** FMT assay using pulmonary fibrosis patient-derived fibroblasts. The upper panel shows the overall procedure for FMT assay, and the low panel indicates the quantified data from high-content analysis (HCA). Left axis and blue dots indicate the percentage of inhibition (PIN) of TGF-β1-mediated α-SMA expression in patient fibroblasts by NP-011 treatment, and the right axis and orange dots indicate the percentage of remaining cells after NP-011 treatment in patient fibroblast. Data are presented as means ± SD from three independent experiments. *p < 0.05, **p < 0.01 (ANOVA).

Given the presence of multiple cell types in AOs, we investigated whether AOs are a useful in vitro model for studying PF. To evaluate the capacity of AOs to respond to a fibrogenic stimulus, we treated AOs with 25 ng/ml TGF-β1 for 72 h and analyzed fibrotic changes at both the transcript and protein levels. We found that treatment of AOs with TGF-β1 significantly induced extracellular matrix (ECM) (*COL1A1* and *COL1A2*), mesenchymal (*VIMENTIN*) and FMT (*α-SMA*, *CTNNB1*, *TWIST1*, and *SNAIL1*)-related genes (Fig. 1A). Furthermore, histochemical and immunofluorescence staining showed an increase in the fibrotic area (Sirius red staining) and expression of α-SMA in AOs treated with TGF-β1 compared to untreated controls (Fig. 1B). At the protein level, TGF-β1 induced increased expression of collagen, fibronectin, phosphorylation of SMAD2 and SMAD3 (henceforth SMAD2/3) and extracellular signal-related kinase (ERK) signaling, as assessed by western blotting (Fig. 1C, D). Altogether, these results suggest that AOs are able to respond fibrogenic stimuli and support the potential of AOs as an in vitro PF model for evaluating therapeutic efficacy of novel drugs.

**Fig. 2:**
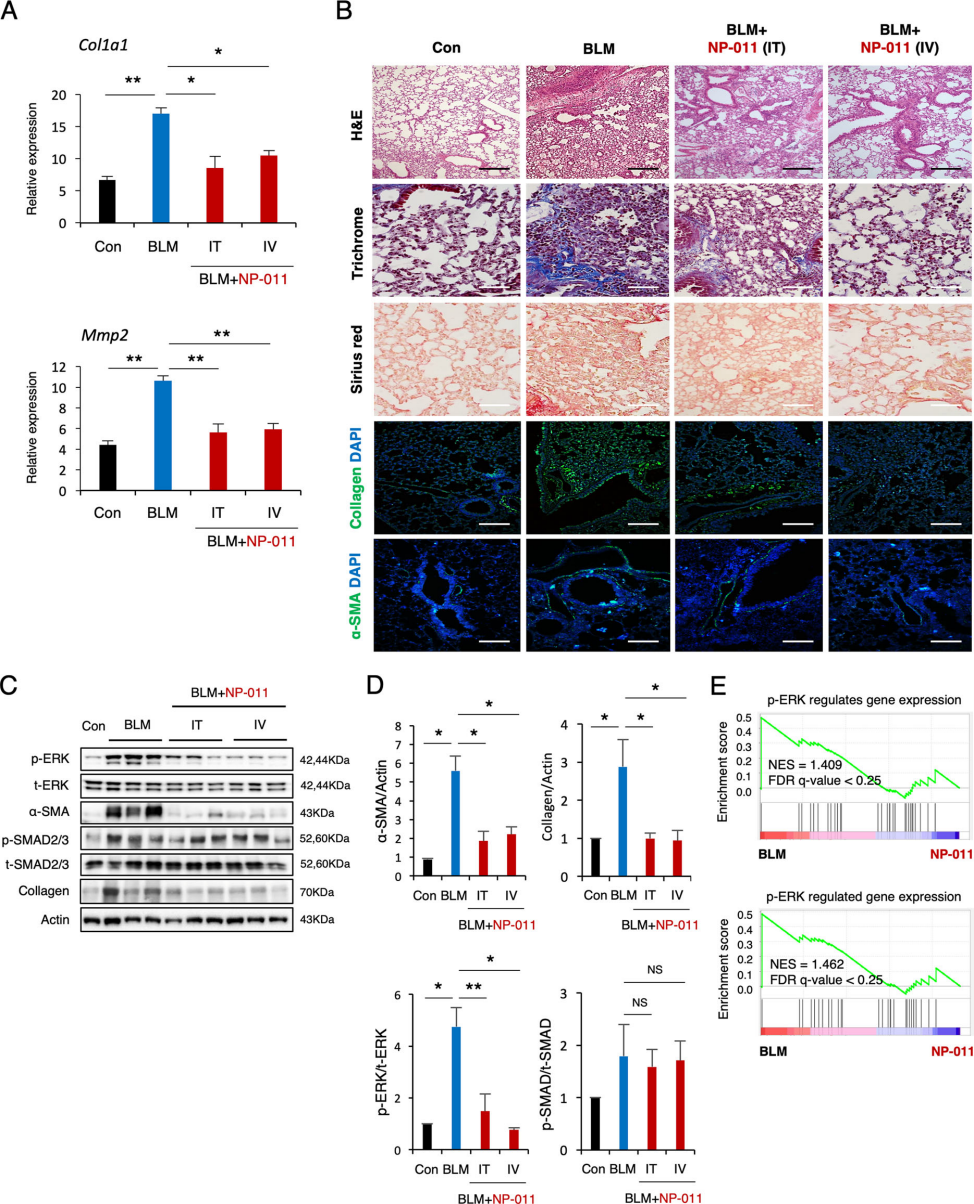
NP-011 reversed existing PF via suppression of ERK signaling. **A** The mRNA levels of *Col1a1* and *Mmp2* in lungs from the indicated groups of mice were detected by qPCR. **B** Representative images show H&E, Masson’s trichrome, Sirius red, Collagen, and α-SMA staining of lung sections from the indicated groups of mice. Scale bars, 100 μm. **C**, **D** Western blotting (**C**) and subsequent quantification of p-ERK, p-SMAD2/3, α-SMA, and Collagen (**D**) in whole-lung homogenates of mice from the indicated groups. Actin was used as a loading control. NS indicates a nonsignificant difference. **E** Two different GSEA data showing the significant suppression of non-canonical ERK signaling gene sets in NP-011-administered group compared to BLM-treated group in PF model. Data are presented as means ± SD; *n* = 10 (control), *n* = 11 (BLM), *n* = 5 (BLM + NP-011 IT), *n* = 7 (BLM + NP-011 IT). *p < 0.05, **p < 0.01 (ANOVA).

Evidence from a recent study suggests an anti-fibrotic property of milk fat globule-epidermal growth factor 8 (MFG-E8)^21–23^, and our collaborator, NEXEL Co., Ltd., recently developed several modified forms of MFG-E8 protein, including NP-011 for evaluation of their anti-fibrotic power compared to MFG-E8. The therapeutic efficacy of NP-011 was tested in our TGF-β1-induced fibrosis AO model. Quantitative reverse transcription PCR (RT-qPCR) demonstrated that increased transcript levels of the ECM (*COL1A1* and *COL1A2*) and FMT (*α-SMA*, *CTNNB1*, *TWIST1*, and *SNAIL1*) markers in fibrotic AOs were significantly reduced by NP-011 (500 ng/ml) treatment (Fig. 1A). Sirius red and immunofluorescence staining revealed that elevated expression of collagen and α-SMA in TGF-β1-treated AOs were markedly diminished by NP-011 treatment (Fig. 1B). In addition, significant reduction of collagen and fibronectin in NP-011-treated AOs was observed by western blot (Fig. 1C, D). We further determined whether the anti-fibrotic effects of NP-011 against TGF-β1-induced fibrosis in the AO model are mediated by canonical or non-canonical SMAD pathways. Interestingly, we found that NP-011 suppressed the phosphorylation of non-canonical ERK in fibrotic lung tissues but had less inhibitory effect on phosphorylation of canonical SMAD2/3 (Fig. 2C, D). The anti-fibrotic effect via suppression of ERK signaling by NP-011 was also recapitulated in human pAECs (Fig. 1E, F). Furthermore, NP-011 treatment significantly inhibited the TGF-β1-induced FMT in 2 different IPF patient-derived fibroblast (Fig. 1G). These results suggest that the hPSC-derived multicellular AOs serve as a model of alveolar tissues and PF pathology and may facilitate the discovery of effective treatments for this disease.

### NP-011 reverses existing PF in mice

We next sought to determine whether hPSC-derived AOs could be used as a platform for predicting or validating drug efficacy in preclinical in vivo animal models. In order to validate the therapeutic effects of NP-011 in vivo, we first observed the development of PF in mice after treatment with BLM (3 mg/kg) to determine the optimal timing of the administration of NP-011 for anti-fibrotic evaluation (Supplementary Fig. 2A). Levels of TGF-β1 pro-inflammatory cytokine and expression levels of fibrosis-related genes (*Col1a1*, *Mmp12*, and *Il-6*) were increased on day 3 after BLM treatment, exhibited considerable enhancement on day 7, and maintained a stable plateau until day 14 (Supplementary Fig. 2B, C). Histochemical analysis also showed a similar temporal increase of fibrotic areas and fibrotic markers (collagen and α-SMA; Supplementary Fig. 2D). Based on these observations, mice challenged with BLM were intravenously treated with two different doses of NP-011 (80 and 160 μg/kg) upon reaching a moderate stage of PF (day 5 post-BLM treatment). Importantly, BLM-induced fibrotic features were obviously alleviated at both the transcript and protein levels after NP-011 administration (Supplementary Fig. 3); however, there were no significant differences in the reduction of fibrotic area and fibrosis markers between the two doses of NP-011. We further compared the therapeutic efficacy of IV and IT administration of NP-011 to determine optimal delivery route. Both administration routes led to a similar reduction in expression levels of fibrotic markers as well as fibrotic area via suppression of ERK signaling, but neither inhibited the phosphorylation of canonical SMAD2/3 (Fig. 2A–D). The therapeutic role of NP-011 via suppression of ERK signaling was further confirmed by two different GSEA, which showed suppression of non-canonical ERK signaling gene sets in NP-011-administered group compared to BLM-treated group (Fig. 2E). Furthermore, transcriptomic analysis of RNA-Seq revealed the potential target genes associated with anti-fibrotic effects of NP-011 on the PF (Supplementary Fig. 4). These findings suggest the predictive value of the in vitro fibrosis AO model for identification of the preclinical therapeutic efficacy of novel drugs for PF.

### Anti-fibrotic effects of NP-011 are comparable to those observed following administration of PFD and Nib

**Fig. 3:**
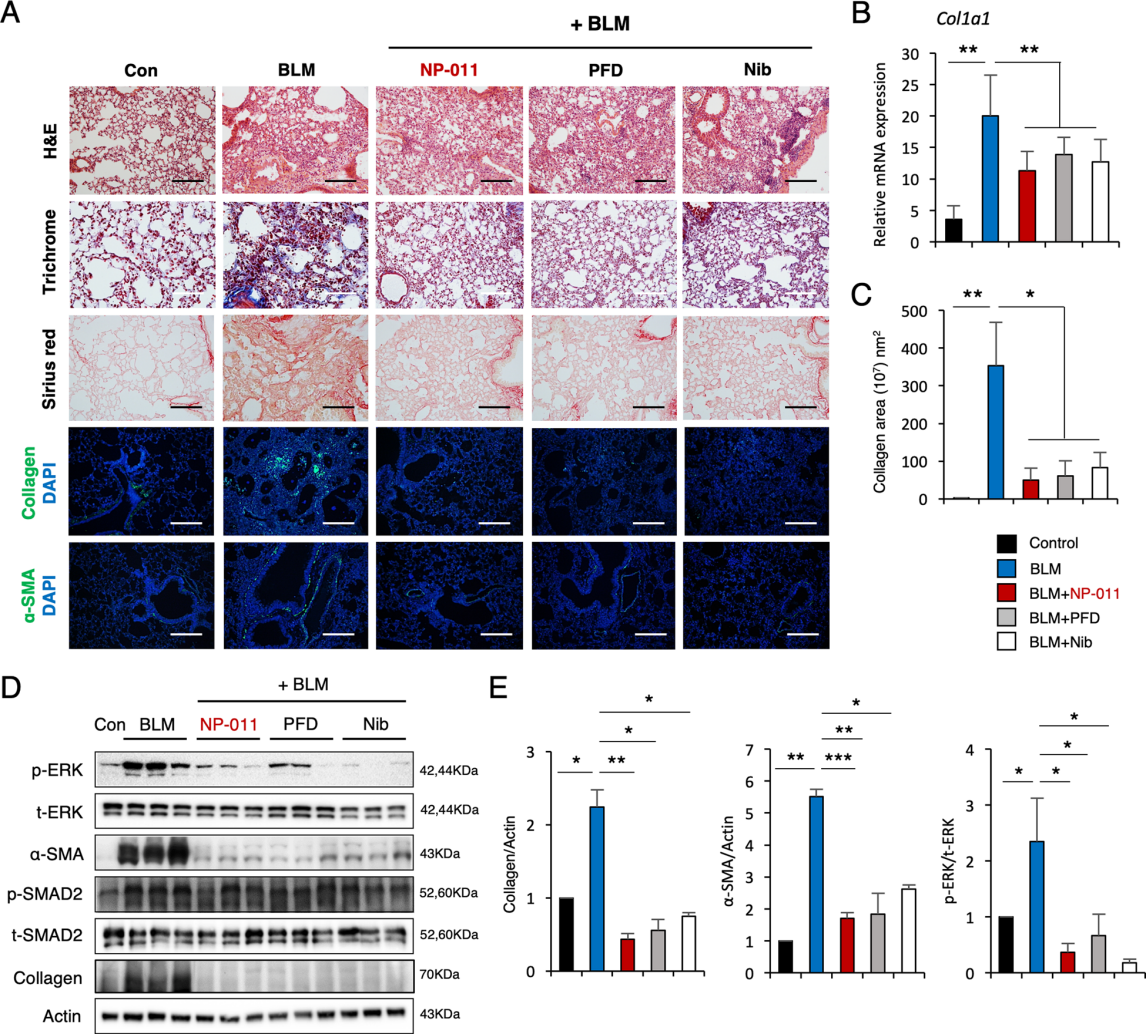
Comparison of the therapeutic effects of NP-011, PFD, and Nib on BLM-induced PF in mice. **A** H&E, Masson’s trichrome, and Sirius red staining of representative lung sections from each group of treated mice. The effects of NP-011, PFD and Nib on the expression of collagen and α-SMA in lung tissues of BLM-induced PF in mice. Scale bars, 100 μm. **B** qPCR analysis for *Col1a1* expression. **C** Quantification of collagen-positive areas as measured by confocal microscopy. **D** Whole lungs from control or BLM-challenged mice treated with NP-011, PFD, and Nib were used for Western blotting using p-ERK, Collagen, α-SMA, and a loading control (Actin). **E** Quantification of p-ERK, Collagen, and α-SMA. Data are presented as means ± SD; *n* = 6 (control), *n* = 8 (BLM), *n* = 7 (BLM + NP-011), *n* = 6 (BLM + PFD), *n* = 8 (BLM + Nib). *p < 0.05, **p < 0.01 (ANOVA).

As NP-011 was found to exert anti-fibrotic effects in a mouse model of PF in vivo, we compared the anti-fibrotic effects of NP-011 with those of PFD and Nib, which are currently approved by the US Food and Drug Administration for treatment of human IPF. Histochemical analysis demonstrated that administration of either of these drugs resulted in effective reduction of PF in mice compared with the untreated control mice (Fig. 3A). Immunofluorescence staining confirmed the decrease in collagen deposition as well as the expression of α-SMA (Fig. 3A). Correspondingly, quantification of collagen transcripts and fibrotic area showed a significant decrease in the lung tissues treated with either NP-011, PFD, or Nib compared with the untreated control mice (Fig. 3B, C). Furthermore, western blot analysis demonstrated a significant reduction of collagen and α-SMA levels in NP-011-, PFD-, and Nib-treated mice, and these effects were mediated by suppression of non-canonical ERK signaling (Fig. 3D, E). These results clearly demonstrate that NP-011 accelerates fibrosis resolution comparable to that induced by administration of PFD and Nib.

### NP-011 facilitates macrophage-mediated collagen uptake in lung fibrosis

**Fig. 4:**
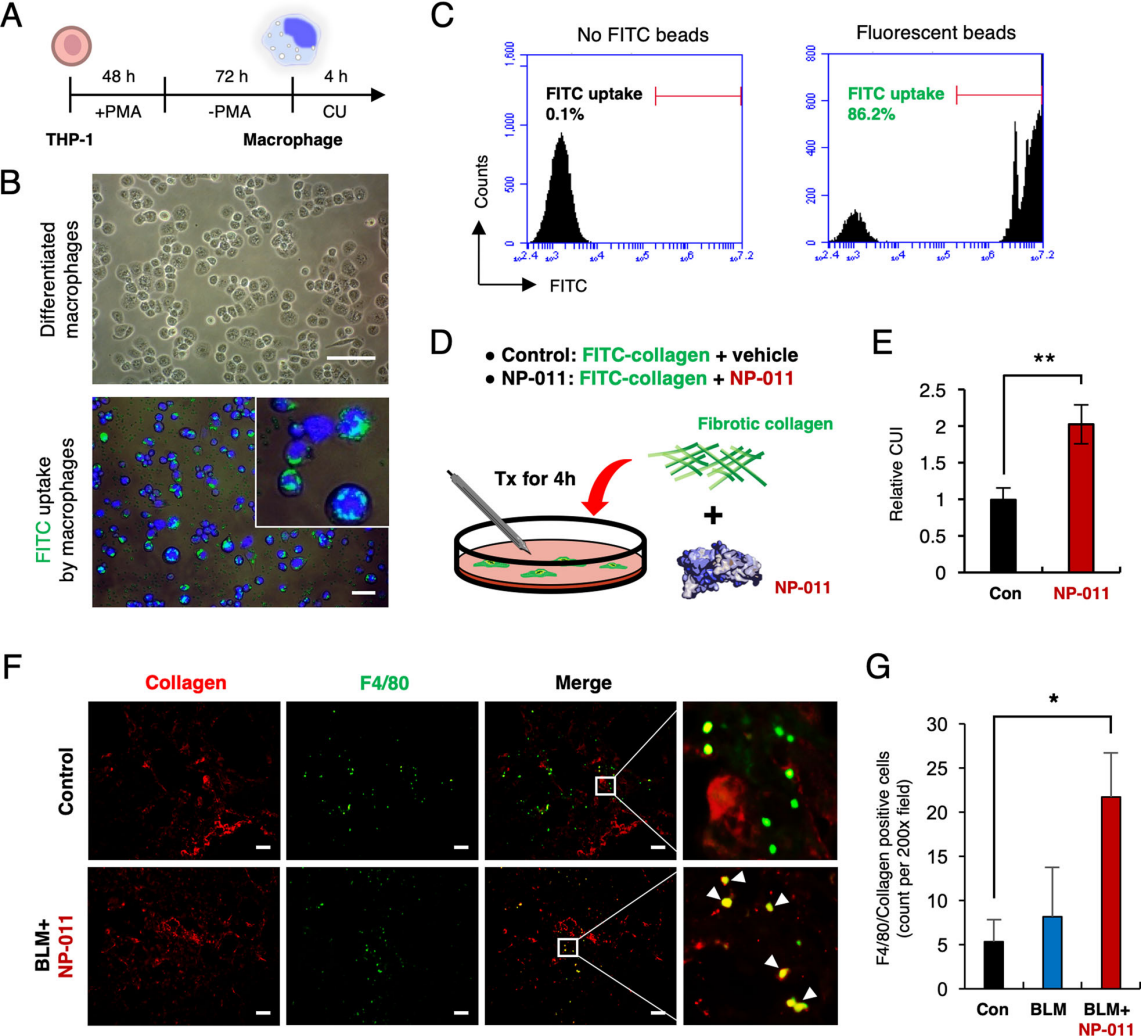
NP-011 facilitates macrophage-mediated collagen uptake in lung tissues. **A** Overall schematic schedule for macrophage differentiation from monocyte by treatment of PMA. **B** Representative images for differentiated macrophages (upper panel) and FITC-labeled beads uptake by differentiated macrophages (lower panel). Scale bar, 10 μm. **C** Flow cytometry results showed that the FITC beads were uptaken by differentiated macrophages. **D** Graphical description of fibrotic collagen uptake assay by differentiated macrophages in the absence and presence of NP-011. **E** Quantitative analysis of the relative CUI (collagen uptake index) for control and NP-011 treatment. Bars represent the means ± SD from three replicates in each group. **P < 0.01 (Student *t*-test). **F** Representative images of immunostaining for mouse collagen and mouse F4/80 in the lung tissues of BLM-induced PF model and NP-011 administrated BLM-induced PF model. Scale bar, 50 μm. **G** Quantification of Collagen^+^F4/80^+^ cells in the lung tissues. The positive cells were counted in three non-repetitive fields of each stained section. Data presented as means ± SD (*n* = 3 mice per group). *p < 0.05 (Student *t*-test).

Elimination of accumulated collagen by immune response is another key factor in resolving fibrosis as seen in PF mouse model^24^. In order to assess this, THP-1 monocytes were differentiated into macrophages by PMA treatment (Fig. 4A), and the phagocytic capability of differentiated macrophages was confirmed by fluorescence and flow cytometry analysis (Fig. 4B, C). Collagen uptake assay revealed that green fluorescence-labeled collagen was significantly up-taken by differentiated macrophages in the presence of NP-011 unlike the control culture (Fig. 4D, E). In fact, the engulfment of collagen by macrophages was demonstrated by immunostaining for collagen and F4/80 after NP-011 administration in BLM-induced PF model (Fig. 4F, G). Taken together, these results suggest that NP-011 could not only reverse fibrosis by inhibiting of mesenchymal transition in lung fibrosis, but also resolve fibrosis by recruiting macrophages in the collagen rich regions of fibrosis to facilitate uptake of deposited collagen in the lung.

## Discussion

Previous studies reported the hPSC-derived lung organoids that represent different respiratory compartments and utilized these models for gaining a deeper understanding of cell-to-cell communications during early lung development and pathogenesis of alveolar diseases, such as Hermanski-Pudlak syndrome and Bochdalek congenital diaphragmatic hernia^11–15,25^. However, the application of hPSC-derived lung organoids that recapitulate the complexity and function of the native lung tissue for disease modeling and drug screening has remained challenging. Here, we describe a protocol for the generation of multicellular AOs, which contain both functional AECs and mesenchymal support cells derived from hPSCs. Our method is based on optimized stepwise direct hPSC differentiation via mimicking of developmental cues in a temporally controlled manner^6,26^. Notably, we showed for the first time that hPSC-derived AOs treated with TGF-β1 exhibited significant fibrogenic responses, which were effectively ameliorated by a drug candidate (NP-011). Furthermore, we observed the anti-fibrotic effects of NP-011 in an in vivo mouse model of PF, underscoring the translational potential of hPSC-derived AOs as a robust in vitro system for respiratory disease modeling and drug testing.

Human MFG-E8 consists of epidermal growth factor-like domain that bears an arginine-glycine aspartate (RGD) motif, followed by two C domains (C1 and C2). MFG-E8 modulates inflammatory responses by RGD motif-mediated binding to immune cells and engulfs apoptotic cells by recognizing phosphatidylserine via C2 domain. Although anti-fibrotic and anti-inflammatory functions of MFG-E8 are well defined in several in vitro and in vivo experimental systems, including myocardial infarction and skin and liver fibrosis^21–23,27^, it is unclear how MFG-E8 is responsible for the anti-fibrotic effects. Thus, we assumed that the roles of the C1 and C2 domains of MFG-E8 might be different, and modulation of these two domains could improve the anti-fibrotic effect of MFG-E8 protein. Our collaborator newly synthesized the C2 domain-truncated form of MFG-E8 (NP-011), which expected to have the enhanced anti-fibrotic power in a fibrosis model compared to a full length of MFG-E8 and the C1 domain-truncated form (NP-012). Based on these findings, we tested the anti-fibrotic effects of NP-011 in both the in vitro AO system and the in vivo PF model. Our study revealed that NP-011 effectively reverses fibrosis via inhibition of ERK signaling, consistent with effects of administration of PFD and Nib. Although these two medications are the only US Food and Drug Administration-approved drugs for the treatment of IPF, these drugs do not work for all patients^3,4^. Therefore, NP-011 may provide a new alternative for IPF patients who do not respond to existing drugs and may enhance response in combination with PFD or Nib as well.

Besides the anti-fibrotic effects of NP-011 for ameliorating PF, NP-011 might have clinical benefits in comparison with MFG-E8 by truncation of C2 domain. First, MFG-E8 contains an amyloid fragment (known as medin), which is positioned within C2 domain^28^. Medin is aortic medial amyloid that occurs in people older than 60 years, and accumulated medin is engaged in Alzheimer’s disease and type 2 diabetes^29^. Therefore, through the structural truncation of C2 domain from MFG-E8, NP-011 might be free for concerning from side effects that possibly cause Alzheimer’s disease and diabetes upon repeated administrations into patients. Second, NP-011 might exhibit better binding affinity against collagen by removing glycosylation site of MFG-E8. MFG-E8 contains glycosylation sites in C2 domain. Thus, NP-011 has no glycosylation that might impede the binding of discoidin domain (C1 domain) of MFG-E8 to accumulated collagen in PF. The well-known discoidin domain receptor (DDR) has discoidin domain, stalk region, and transmembrane domain in its structure^30^. Although the mechanism of collagen binding of DDR has not been identified, the binding of discoidin domain of DDR to collagen has been reported^31^. Interestingly, the discoidin domain of DDR has no glycosylation, suggesting that discoidin domain of NP-011 might show better similarity to discoidin domain of DDR than MFG-E8, possibly facilitating the efficient binding to accumulated collagen in the lung.

While the promising data presented in this study and multicellular composition of hPSC-derived AOs is a major advantage, one of the main limitations of this system, similar to other hPSC-derived organoid systems, is that the AOs remain incomplete as they lack of key components of the native alveolar tissue, including macrophages and microvasculature. Macrophages are remarkably plastic cells that can transform from the pro-inflammatory M1 phenotype to the anti-inflammatory M2 phenotype and vice versa^32^. Internal and external stimuli induce dynamic changes in macrophage phenotype, which is closely associated with either exacerbation or prevention of IPF progression^33,34^. Furthermore, a fine mesh of capillaries wraps around each alveolus and covers about 70% of its surface area for gas exchange. Therefore, the development of AOs closer to the native alveolar tissue architecture and function by the incorporation of these missing components will provide more accurate and robust in vitro platform for studying PF and associated therapeutics. Although the ability to differentiate hPSCs into three germ layer lineages presents great promise for the development of various organoid models that recapitulate the complexity and functions of in vivo tissues, the variations in the differentiation potential among hPSC lines due to genetic background, culture conditions and differentiation protocols may interfere with organoid quality, reproducibility and faithfulness. Thus, the field needs a standardized differentiation protocol with gold standard hPSC lines, which may able to diminish off-target cells and generate highly homogenous organoids.

## Materials and methods

### Maintenance of hPSCs

Human PSCs (CHA15 and iPS-NT4-S1) were kindly provided by CHA University, South Korea and maintained as previously described^35^. Briefly, the cells were cultured under xeno-, serum- and feeder-free conditions using E8 medium (STEMCELL Technologies) on dishes coated with vitronectin (STEMCELL Technologies). Cells were subcultured at 80% confluency and passaged every 5 days by mechanical dissociation. All cells were incubated at 37 °C in a humidified atmosphere with 5% CO_2._

### Cell cultures

Human pAECs were purchased from Sciencell Research Laboratories, Inc. and cultured in alveolar epithelial cell medium (#3201, Sciencell, Carlsbad, CA, USA) at 37 °C, 5% CO_2_. Human pAECs were treated with 5 ng/ml TGF-β1 and 500 ng/ml NP-011 for 48 h and harvested for further analysis.

### Stepwise differentiation of hPSCs into AECs

A stepwise direct AEC differentiation was performed as previously described^6,36^. Briefly, undifferentiated hPSCs were dissociated and then plated in dishes coated with vitronectin at a density of 1 × 10^5^ cells/cm^2^. After overnight incubation, AEC differentiation was initiated with exposure to stepwise induction medium.

### Generation of multicellular AOs from hPSCs

Generation of multicellular AOs was performed by combination of previously reported protocols with minor modifications^36–38^. Briefly, the AEC cultures were dissociated on day 21 of AEC differentiation with 0.4 U/ml collagenase B (Roche) for 2 h in a 37 °C incubator, followed by treatment with cell dissociation buffer (Gibco) for 10 min in a 37 °C water bath. The single-cell suspension was then passed through a 70-μm cell strainer (BD Bioscience) and seeded into 96-well round-bottom plates (Corning, 5 × 10^4^ cells per well) containing AEC maturation medium supplemented with 10 μM Rho-associated protein kinase inhibitor (STEMCELL Technologies). After distribution, 1:15 diluted Matrigel (150 μl per well) was added into each well to improve adhesion between cells. The plates were centrifuged at 450 × *g* for 5 min and incubated overnight to allow aggregation at 37 °C in a humidified atmosphere with 5% CO_2_. After overnight incubation, the aggregates were transferred to 6-well low-attachment plates (Corning) containing fresh AEC maturation medium and cultured for 6 days to establish AOs.

### Mouse model for bleomycin-induced lung fibrosis and drug treatments

All animal experiments were approved and followed the regulations of the Institutional Animal Care and Use Committee of Kangwon National University (IACUC NO. KW-180913-1). Sample size estimation was calculated by IACUC and online free software (Open Epi, http://www.openepi.com/OE2.3/Menu/OpenEpiMenu.htm). Male mice (C57BL/6), 8 to 10 weeks of age were purchased from the Dooyeol Laboratory. Lung fibrosis was induced by intratracheal (IT) administration of 50 μl of bleomycin (BLM) (3 mg per kg body weight, mg/kg) and the mice were randomly grouped in a blinded manner. On day 5 after BLM administration, mice were given an intravenous (IV) or IT injection of NP-011 (80 and 160 μg/kg), PFD (300 mg/kg) and Nib (60 mg/kg). On day 3 after drug administration, mice were sacrificed and lung tissues were examined.

### RNA extraction and quantitative real-time PCR (qPCR)

Total RNA was extracted from mouse lung tissues, AOs, pAECs and undifferentiated hPSC cultures using an RNeasy Mini kit (Qiagen, Duesseldorf, Germany) and complementary DNA was synthesized using TOPscrip^TM^ RT DryMIX (Enzynomics, Daejeon, Korea). PCR amplification was performed using a Step One Plus real-time PCR system (Applied Biosystems, Warrington, UK) with TOPreal^TM^ qPCR 2X PreMIX (Enzynomics). All the mRNA expression was normalized to an internal control GAPDH. The primer sequences for human and mouse genes are listed in Supplementary Tables 1 and 2, respectively.

### Immunohistochemistry for paraffin section

AOs and lung tissues were paraffin-embedded, sectioned at a thickness of 5 μm and mounted on slide glass. The slides were rinsed in xylenes and with decreasing concentration (100% to 80%) of ethanol to deparaffinize and rehydrate. The slides were subjected to hydrated autoclaving using an automated antigen-retrieval a citrate buffer (0.01 M sodium citrate, pH 6) for 121 °C for 1 min and cooled for 30 min before immunostaining. They were then treated with Dako Real Peroxidase-blocking solution for 20 min at room temperature (RT) and washed with cold phosphate-Buffered Saline with 0.1% Tween 20 (PBST) for 10 min at RT. The slides were blocked with 10% normal goat serum for 1 h at RT and incubated with primary antibodies in blocking buffer overnight at 4 °C. The next day, goat anti-mouse IgG (H + L) Cross-Adsorbed Secondary Antibody, Alexa Fluor 488 (Invitrogen, A11001) secondary antibody was applied for 1 h at RT in the dark. Eventually, the slides were washed with PBST (0.1% tween) and covered with Fluoroshield^TM^ mounting medium with 4’, 6-diamidino-2-phenylindole (DAPI). IHC images were captured under a fluorescence microscopy (IX-51, Olympus). To evaluate anti-fibrotic effect of NP-011 in vivo, three mice were used in each group and data were obtained in at least three fields. The positive area of collagen of total area were measured by using LSM880 with Airscan.

### Immunofluorescence staining for frozen AO sections

AOs were fixed with 4% paraformaldehyde for 24 h at RT, followed by dehydration using sucrose solution of a gradual gradient series. The fixed AOs were then embedded in Tissue-Tek^®^ cryomold (Sakura finetek) covered with Tissue-Tek^®^ OCT^TM^ Compound (Sakura finetek) rocking at 4 °C overnight and stored in −80 °C. Frozen AO blocks were sectioned into 8 μm cryosections, which were carefully mounted onto silane-coated micro slides (Muto Pure Chemicals) and stored in −80 °C. The sections were blocked by 10% donkey serum (Jackson ImmunoResearch Laboratories) in PBST for 1 h at RT and probed with primary antibodies (EPCAM, CPM, HOPX, SFTPC, AQP5, T1α and VIMENTIN) in 1% donkey serum in PBST at 4 °C overnight. The next day, the sections were rinsed with PBST for 5 min, followed by incubation with secondary antibodies for 30 min at RT. The sections were counterstained using Fluoroshield^TM^ with DAPI histology mounting medium (Sigma-Aldrich). IHC images were captured under a fluorescence microscopy (IX-51, Olympus). Primary antibodies were omitted in control immunohistochemical staining. The antibodies are listed in Supplementary Table 3.

### Hematoxylin and eosin, Sirius red, and Masson’s trichrome staining

Paraffin sections of AOs and lung tissues were deparaffinized by sinking in xylene and sequentially rehydrated through a gradual concentration series, starting from 100% ethanol to 70% ethanol and ending with deionized water. The sections were stained to nucleus using Hematoxylin (BBC biochemical) and rinsed by water, followed by a short exposure of acidic ethanol. The sections also were exposed by Eosin targeting to cytoplasm and dehydrated through a sequential concentration change, starting from 70% ethanol to 100% ethanol. After exposure to xylene three times, the sections were mounted with Permount mounting medium (Fisher Scientific). The fibrotic areas in AOs and lung sections were detected using Masson’s trichrome (Empire Genomics, #BPK2916, USA) and Sirius red staining (Abcam, ab150681, UK) according to the manufacturer’s instructions.

### Western blot analysis

Protein extracted from mouse lung tissues, AOs and human pAECs were lysed in protein lysis buffer and quantified using the bicinchoninic acid protein assay. The 20 μg of protein were separated by sodium dodecyl sulfate polyacrylamide gel electrophoresis (8–15%) gel and then transferred to polyvinylidene fluoride membranes. Nonspecific binding proteins were blocked with 5% skim milk for 1 h at RT. The membranes were incubated with primary antibodies against anti-phospho-p44/42 MAPK, anti-p44/42 MAPK, anti-alpha smooth muscle actin (α-SMA), anti-phospho-SMAD2/3, anti-SMAD2/3, anti-Collagen1A and anti-Fibronectin overnight at 4 °C. The chemiluminescence signal was scanned with ChemiDOC^TM^ imaging system (Bio-Rad Laboratories, Hercules, CA, USA). The antibodies are listed in Table S3.

### Enzyme-linked immunosorbent assay (ELISA)

Concentration of TGF-β1 in bronchoalveolar lavage fluid (BALF) was measured by ELISA kits (R&D, Minneapolis, MN, USA) according to the manufacturer’s instructions.

### RNA-sequencing and analysis

Total RNA was extracted from lung tissues of control, BLM-administered and NP-011-administered groups using Trizol reagent (Invitrogen). RNA quality was assessed by Agilent 2100 Bioanalyzer using the RNA 6000 Nano Chip (Agilent Technologies, Amstelveen, The Netherlands), and RNA quantification was performed using ND-2000 spectrophotometer (Thermo Inc., DE, USA). Triplicate samples were prepared for each group. For control and test RNAs, the construction of library was performed using QuantSeq 3’mRNA-Seq Library Prep Kit (Lexogen, Inc., Austria) according to the manufacturer’s instructions. In brief, each 500 ng total RNA were prepared and an oligo-dT primer containing an Illumina-compatible sequence at its 5’end was hybridized to the RNA and reverse transcription was performed. After degradation of the RNA template, second strand synthesis was initiated by a random primer containing an Illumina-compatible linker sequence at its 5’end. The double-stranded library was purified by using magnetic beads to remove all reaction components. The library was amplified to add the complete adapter sequences required for cluster generation. The finished library is purified from PCR components. High-throughput sequencing was performed as single-end 75 sequencing using NextSeq 500 (Illumina, Inc., USA). To analyze RNA-Seq, these samples were sent to the BGI Tech Solutions Company (Hongkong). Gene Set Enrichment Analysis (GSEA) was conducted using GSEAv17 (Broad Institute, Cambridge, MA, USA).

### Fibroblast to myofibroblast transition (FMT) assay

FMT assay was performed by Charles River Laboratories International. Briefly, lung fibroblast from two different Idiopathic PF (IPF) donors were seeded onto culture plates. IPF fibroblasts were cultured with eight different concentrations of NP-011 and then treated with TGF-β1. α-SMA-positive cells were assessed by high-content analysis (HCA).

### THP-1 differentiation and fluorescent bead phagocytosis assay

THP-1 cells were purchased from ATCC and cultured in RPMI-1640 (Gibco, 31800-022) containing 10% of heat inactivated fetal bovine serum (Gibco, 16000-044) and 50 μM β-mercaptoethanol in 37 °C, 5% CO_2_ incubator. THP-1 cells were differentiated into macrophages using 200 ng/ml of phorbol 12-myristate 13-acetate (PMA, Sigma, P8139) for 48 h followed by 72 h in PMA-free medium. In phagocytosis assay, THP-1 cells were seeded at 1 × 10^5^ cells/cm^2^. Carboxylate-Modified Microspheres, 2.0 μm, yellow-green fluorescent beads (Invitrogen, F8827) were washed in THP-1 cell culture media and resuspended at a final dilution of 1:500 in serum-free RPMI-1640. Cells were incubated with fluorescent beads for 4 h in 37 °C, 5% CO_2_ incubator. Cells were detached with TrypLE (Gibco, 12604-021) and measured by flow cytometry (Accuri C6 Plus).

### Collagen uptake assay

THP-1 cells were differentiated into macrophages by 200 ng/ml of PMA treatment and incubated with 50 μg/ml of fluorescein isothiocyanate (FITC)-conjugated type I collagen (Anaspec, AS-85111) in serum-free media with or without 15 μg/ml of NP-011 for 4 h at 37 °C. After 4 h, differentiated macrophages were washed several times to remove un-ingested collagen and fixed for 20 min with 4% paraformaldehyde. The cells were counterstained with DAPI. Internalization of collagen was quantified by fluorescence microscopy and expressed as a collagen uptake index (CUI: Number of cells with ingestion of FITC-collagen divided by the total number of cells counted).

### Statistical analysis

Values for all measurements are presented as means ± standard deviation (SD). Student’s *t*-test was used for comparisons between two groups. One-way ANOVA was performed for multigroup comparisons. All test utilized one-sided methodology. A *P*-value of <0.05 was considered statistically significant.

## Supplementary information

Supplementary Figure Legends

Supplementary Table and Legends

Supplementary Figure 1

Supplementary Figure 2

Supplementary Figure 3

Supplementary Figure 4
