# Supplementary material for: Human pluripotent stem-cell-derived alveolar organoids for modeling pulmonary fibrosis and drug testing
Source: Cell Death Discov. 2021 Mar 15;7:48. doi: 10.1038/s41420-021-00439-7 (PMC7961057; doi:10.1038/s41420-021-00439-7)
Supplement: Supplementary file 1 — Supplementary Figure Legends [file 41420_2021_439_MOESM1_ESM.docx]

**Supplementary Figure Legends**

**Supplementary** **Fig. 1 Generation of multicellular hPSC-AOs. A** A stepwise induction protocol for generating multicellular AOs from hPSCs. **B** Representative bright field (BF) and H&E staining images of AOs from day 27. Scale bars, 100 μm. **C** Representative immunofluorescence images in AOs at day 27 stained with AEP (EPCAM, HOPX and CPM, red), AEC1 (AQP5 and T1α, green), AEC2 (SFTPC, red) and mesenchymal cell (VIM) markers. Scale bars, 100 μm. DAPI (blue) is used to visualize nuclei. **D** mRNA expression levels of each specific markers in the undifferentiated (UN) hPSCs and AOs (day 27). Data are shown as fold-change relative to undifferentiated hPSCs. Data are presented as means ± SD from three independent experiments. **p*<0.05, ***p*<0.01 (Student *t*-test).DE, definitive endoderm; AFE, anterior foregut endoderm; VAFE, ventral anterior foregut endoderm; ADAE, alveolar and distal airway epithelial cells.

**Supplementary** **Fig. 2 Development of BLM-induced PF in mice.** **A** Study design. Mice were given a single IT instillation of BLM (3 mg/kg). The lung tissues were isolated to prepare lysates and paraffin sections at the indicated days. **B** ELISA assay for TGF-β1 concentration in BALF. **C** The mRNA levels of *Col1a1*, *Mmp12*, and *Il-6* were detected by qPCR. **D** Representative images show Masson’s trichrome, Sirius red, Collagen and α-SMA staining of lung sections from the indicated groups of mice. Scale bars, 100 μm. Data presented as means ± SD (n ≥ 5 mice per group). **p*<0.05, ***p*<0.01 (Student *t*-test).

**Supplementary** **Fig. 3 Comparison of therapeutic effects of NP-011 doses in BLM-induced PF mice. A** On day 5 after the instillation of BLM, mice were given a single IV administration of NP-011 (80 and 160 μg/kg). The lung tissues were isolated to prepare lysates and paraffin sections at day 8 after BLM. The mRNA levels of *Col1a1* and *Mmp2* in lungs from the indicated groups of mice were detected by qPCR. **B** Representative images show Sirius red, Masson’s trichrome, α-SMA and Collagen staining of lung sections from the indicated groups of mice. Scale bars, 100 μm. **C** Whole lung homogenates from control and BLM-challenged mice treated with NP-011 were used for Western blotting using α-SMA and Collagen. Actin was used as loading control. **D** Quantification of α-SMA and Collagen. Data presented as means ± SD (*n* = 5 mice per group). **p*<0.05, ***p*<0.01, ****p*<0.001 (ANOVA).

**Supplementary** **Fig. 4 Potential target genes of NP-011 in the lungs of BLM-induced PF mice.** The graph represents the potential target genes of NP-011 via RNA-Seq analysis in the lungs of BLM-induced PF mice.
