# Supplementary material for: Human pluripotent stem-cell-derived alveolar organoids for modeling pulmonary fibrosis and drug testing
Source: Cell Death Discov. 2021 Mar 15;7:48. doi: 10.1038/s41420-021-00439-7 (PMC7961057; doi:10.1038/s41420-021-00439-7)
Supplement: Supplementary file 2 — Supplementary Table and Legends [file 41420_2021_439_MOESM2_ESM.docx]

**Supplementary Table 1.** Human Primer sequences used for qPCR

| Genes |  | Sequence 5' to 3' | Product size (bp) |
| --- | --- | --- | --- |
| *T1α* | F | TGC GAA AAA TGT CGG GAA GG | 51 |
|  | R | GGC GTA ACC CTT CAG CTC TT |  |
| *SFTPB* | F | GCC ATA CCA CAG GCA ATG CT | 80 |
|  | R | TGC TGC TCC ACA AAT TGC TT |  |
| *SFTPC* | F | CCT TCT TAT CGT GGT GGT GGT | 96 |
|  | R | TCT CCG TGT GTT TCT GGC TCA T |  |
| *CPM* | F | TCC AAG GTG GAA TGC AAG AT | 181 |
|  | R | TCA AAA ACT TGA CCC TTT ACA C |  |
| *HOPX* | F | GCC TTT CCG AGG AGG AGA C | 97 |
|  | R | TCT GTG ACG GAT CTG CAC TC |  |
| *EPCAM* | F | AGA ACC TAC TGG ATC ATC ATT | 101 |
|  | R | CGC GTT GTG ATC TCC TTC TG |  |
| *NKX2.1* | F | AGC ACA CGA CTC CGT TCT CA | 75 |
|  | R | CCT CCA TGC CCA CTT TCT TG |  |
| *VIMENTIN* | F | CCA GGC AAA GCA GGA GTC | 212 |
|  | R | CGA AGG TGA CGA GCC ATT |  |
| *α-SMA* | F | GAC GAA GCA CAG AGC AAA AG | 70 |
|  | R | AGT TGG TGA TGA TGC CAT GT |  |
| *COL1A1* | F | AAG GGT GAG ACA GGC GAA CA | 70 |
|  | R | GAC CCT GGA GGC CAG AGA AG |  |
| *COL1A2* | F | CGG AGG TAT GCA GAC AAC GA | 99 |
|  | R | ACG GGG CTG GCT TCT TAA AT |  |
| *TGF-β1* | F | AGC AAC AAT TCC TGG CGA TA | 90 |
|  | R | CAC AAC TCC GGT GAC ATC AA |  |
| *CTNNB1* | F | AAA ATG GCA GTG CGT TTA | 99 |
|  | R | TTT GAA GGC AGT CTG TCG TA |  |
| *TWIST1* | F | AGC AAG ATT CAG ACC CTC AAG | 145 |
|  | R | ATC CTC CAG ACC GAG AAG G |  |
| *SNAIL1* | F | TTT ACC TTC CAG CAG CCC TA | 73 |
|  | R | GAC AGA GTC CCA GAT GAG CA |  |
| *IL-11* | F | CTG TGG GGA CAT GAA CTG TG | 115 |
|  | R | AGG GTC TGG GGA AAC TCG |  |
| *GAPDH* | F | GGC ATG GAC TGT GGT CAT GA | 87 |
|  | R | TGC ACC ACC AAC TGC TTA GC |  |

**Supplementary Table 2.** Mouse Primer sequences used for qPCR

| Genes |  | Sequence 5' to 3' | Product size (bp) |
| --- | --- | --- | --- |
| *Col1a1* | F | CTG GCG GTT CAG GTC CAA T | 141 |
|  | R | TTC CAG GCA ATC CAC GAG C |  |
| *Mmp2* | F | GCG ATG TCG CCC CTA AAA CAG | 265 |
|  | R | CTG TAT GTG ATC TGG TTC TTG TCC |  |
| *Mmp12* | F | TGG TAT TCA AGG AGA TGC | 69 |
|  | R | GGT TTG TGC CTT GAA AAC |  |
| *Il-6* | F | AGG ATA CCA CTC CCA ACA GAC CT | 141 |
|  | R | CAA GTG CAT CAT CGT TGT TCA TAC |  |
| *Gapdh* | F | GTT GTC TCC TGC GAC TTC A | 184 |
|  | R | GGT GGT CCA GGG TTT CTT A |  |

**Supplementary Table 3.** Antibodies used for immunostaining and Western blot

| Antibodies | Type | Catalog number | MW(KDa) | Manufacturer |
| --- | --- | --- | --- | --- |
| p-ERK | rabbit | #4370 | 42,44 | Cell Signaling |
| t-ERK | rabbit | #4695 | 42,44 | Cell Signaling |
| p-SMAD2/3 | rabbit | #8828 | 52,60 | Cell Signaling |
| t-SMAD2/3 | mouse | sc-133098 | 52,60 | Santa Cruz |
| α-SMA | mouse | sc-53015 | 43 | Santa Cruz |
| Collagen | mouse | sc-293182 | 70,90 | Santa Cruz |
|  | mouse | sc-59772 | 130-140 | Santa Cruz |
| β-Actin | mouse | sc-47778 | 43 | Santa Cruz |
| Fibronectin | mouse | sc-59826 | 220 | Santa Cruz |
| AQP5 | rabbit | PA5-36529 | 36 | Thermo Fisher |
| T1α | rabbit | PA5-82301 | 40-43 | Thermo Fisher |
| CPM | goat | sc-74380 | 51 | Santa Cruz |
| EPCAM | mouse | sc-66020 | 40 | Santa Cruz |
| HOPX | mouse | sc-398703 | 9 | Santa Cruz |
| GAPDH | rabbit | #2118 | 37 | Cell Signaling |
| VIMENTIN | mouse | sc-373717 | 57 | Santa Cruz |
| F4/80 | mouse | sc-377009 | 160 | Santa Cruz |
| SFTPC | rabbit | ab40879 | 21 | Abcam |

MW, Molecular weight
