# Supplementary figures and images for: Human pluripotent stem-cell-derived alveolar organoids for modeling pulmonary fibrosis and drug testing
Source: Cell Death Discov. 2021 Mar 15;7:48. doi: 10.1038/s41420-021-00439-7 (PMC7961057; doi:10.1038/s41420-021-00439-7)

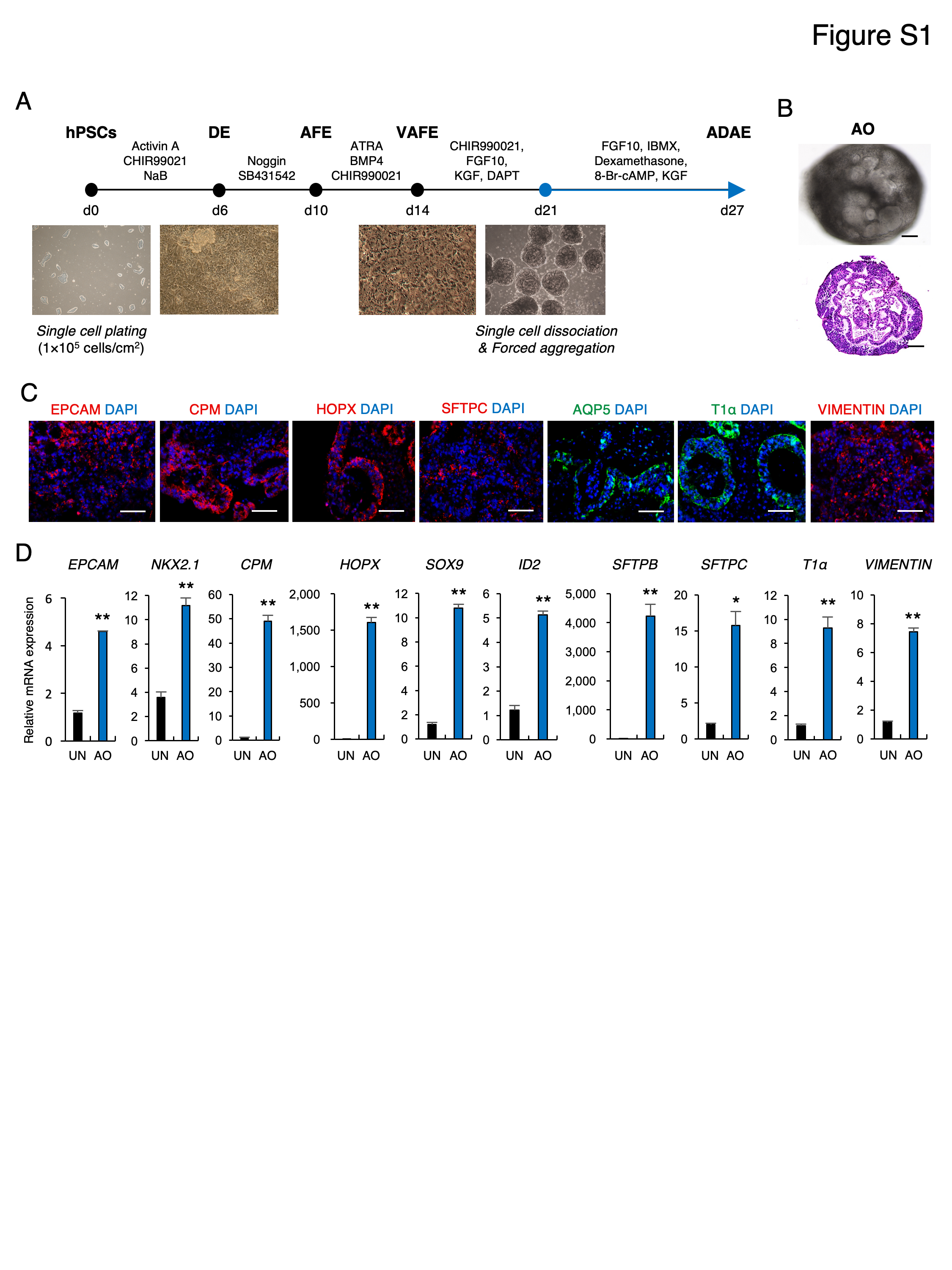

Supplement: Supplementary file 3 — Supplementary Figure 1 [file 41420_2021_439_MOESM3_ESM.png]

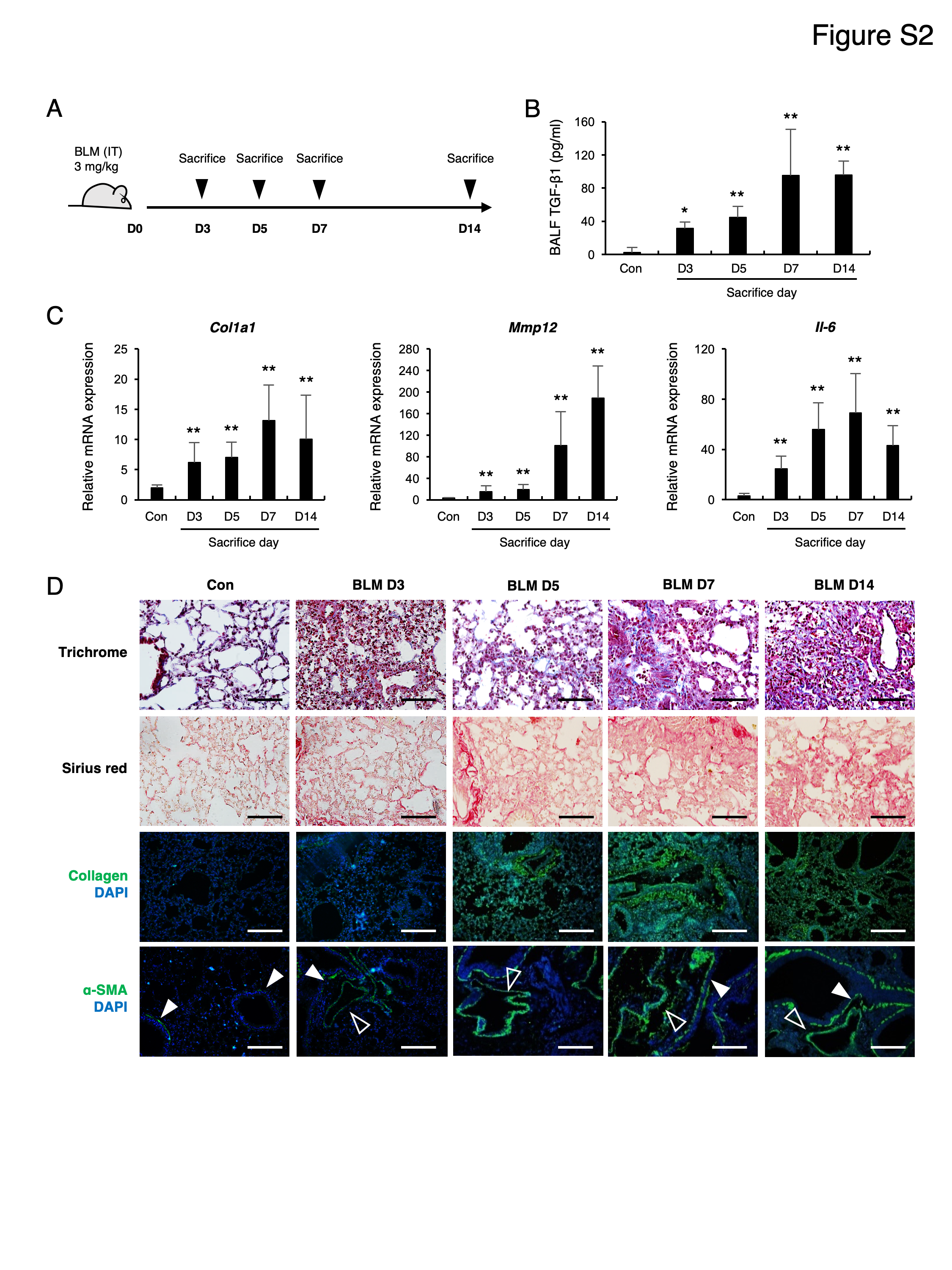

Supplement: Supplementary file 4 — Supplementary Figure 2 [file 41420_2021_439_MOESM4_ESM.png]

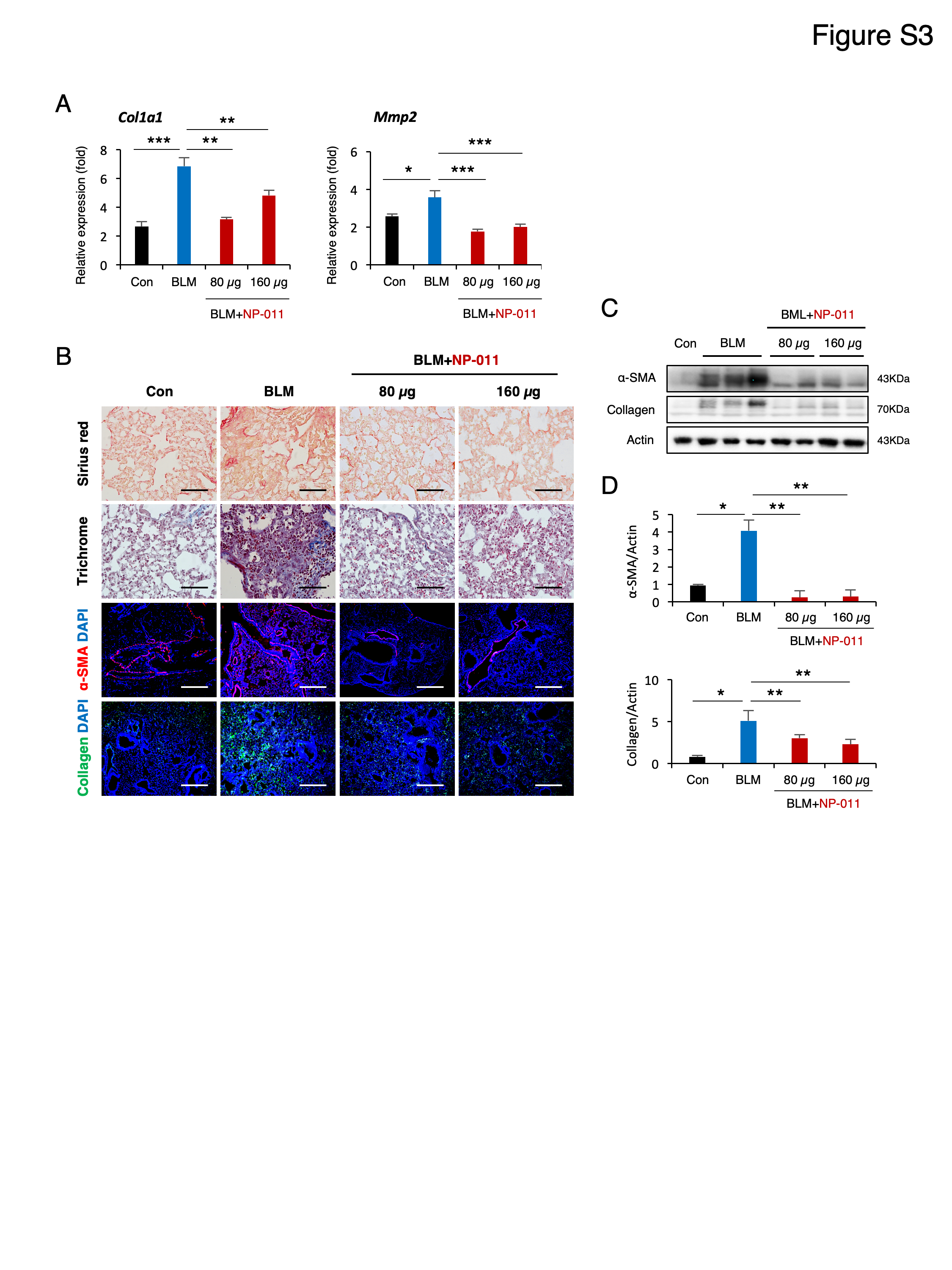

Supplement: Supplementary file 5 — Supplementary Figure 3 [file 41420_2021_439_MOESM5_ESM.png]

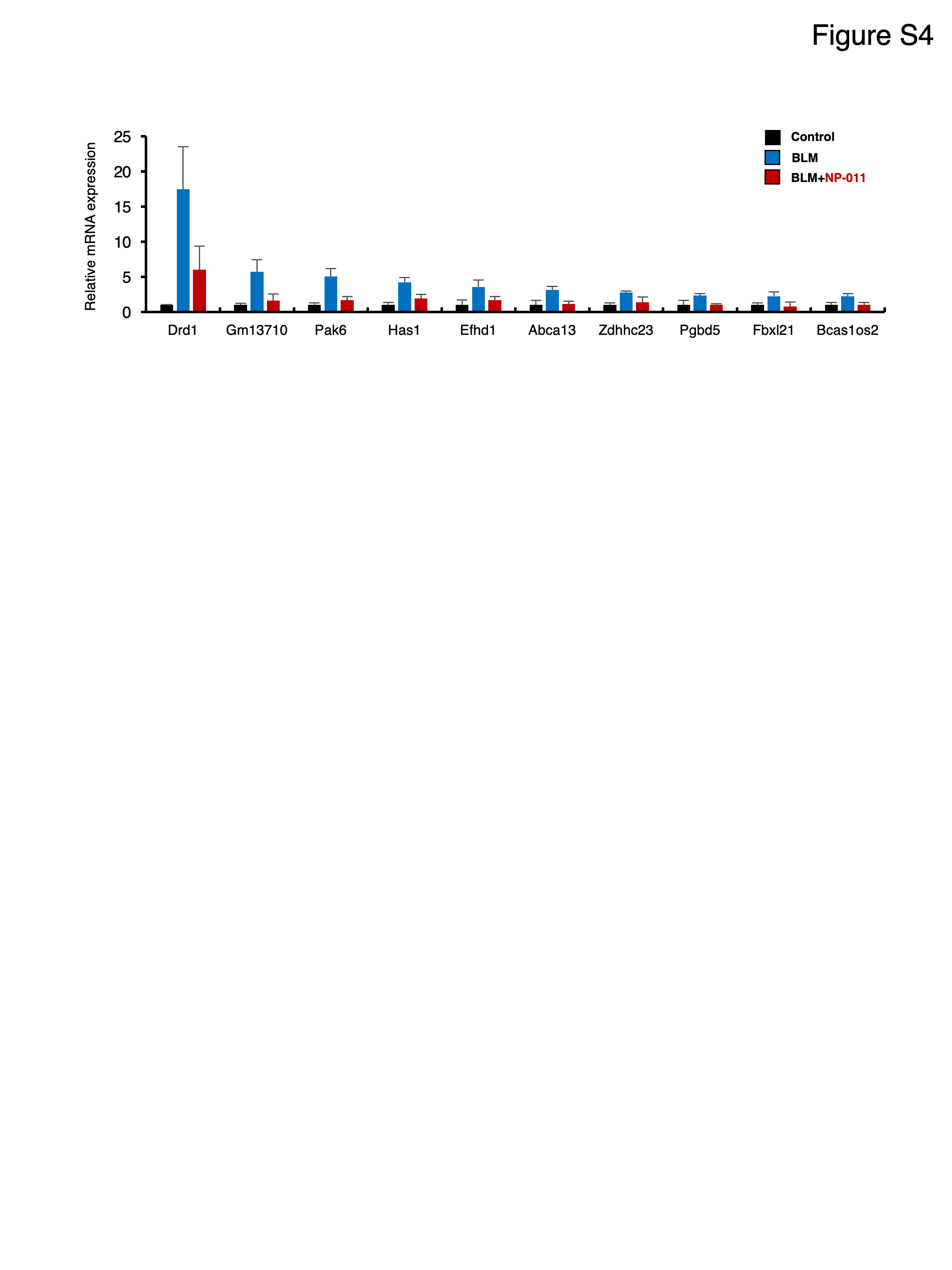

Supplement: Supplementary file 6 — Supplementary Figure 4 [file 41420_2021_439_MOESM6_ESM.png]
